# Conspecific and Heterospecific Plant Densities at Small-Scale Can Drive Plant-Pollinator Interactions

**DOI:** 10.1371/journal.pone.0077361

**Published:** 2013-10-21

**Authors:** Zdeněk Janovský, Michael Mikát, Jiří Hadrava, Eva Horčičková, Kateřina Kmecová, Doubravka Požárová, Jan Smyčka, Tomáš Herben

**Affiliations:** 1 Department of Botany, Faculty of Science, Charles University, Prague, Czech Republic; 2 Secondary grammar school of Znojmo, Znojmo, Czech Republic; 3 Secondary grammar school of Rakovník, Rakovník, Czech Republic; 4 Institute of Botany, Academy of Sciences of the Czech Republic, Průhonice by Prague, Czech Republic; Centro de Investigación y de Estudios Avanzados, Mexico

## Abstract

Generalist pollinators are important in many habitats, but little research has been done on small-scale spatial variation in interactions between them and the plants that they visit. Here, using a spatially explicit approach, we examined whether multiple species of flowering plants occurring within a single meadow showed spatial structure in their generalist pollinator assemblages.

We report the results for eight plant species for which at least 200 individual visits were recorded. We found that for all of these species, the proportions of their general pollinator assemblages accounted for by particular functional groups showed spatial heterogeneity at the scale of tens of metres. This heterogeneity was connected either with no or only subtle changes of vegetation and flowering species composition. In five of these species, differences in conspecific plant density influenced the pollinator communities (with greater dominance of main pollinators at low-conspecific plant densities). The density of heterospecific plant individuals influenced the pollinator spectrum in one case.

Our results indicate that the picture of plant-pollinator interactions provided by averaging data within large plots may be misleading and that within-site spatial heterogeneity should be accounted for in terms of sampling effort allocation and analysis. Moreover, spatially structured plant-pollinator interactions may have important ecological and evolutionary consequences, especially for plant population biology.

## Introduction

Generalist pollinators constitute a major proportion of pollinators (both in terms of species and individuals) in many ecosystems [1], [2], [3]. Additionally, they are involved in the responses of plant-pollinator interactions to ecosystem changes such as the spread of invasive plants [4] and ecosystem restoration [5]. Moreover, the diversity of their visited plant species (i.e. “degree of generalism”) directly influences some key pollinator network characteristics including network asymmetry [6] and the number of cross-links among the network modules [7].

Although generalist pollinator species are characterised by pollinating multiple species of plants, not only can there be specialization among individuals due to flower constancy (e.g. [8]), but the predominant species pollinated by a given generalist species can vary spatially and temporally. Indeed, considerable evidence has accumulated of temporal variation, which commonly is caused by year-to-year or within-season turnover in the spectrum of flowering plant species [3], [9]. However, our knowledge of spatial variability in plant-pollinator interactions involving generalist pollinators is much more scant, and is primarily based on comparisons either at continental scales [10] or among localities several kilometres apart [5]. Thus, the ecological effects of small-scale differences in plant and pollinator spatial distributions as well as species compositions have largely escaped field investigation, despite the predicted importance of such variation in mutualist networks [11]. Therefore, we largely lack empirical data on spatial heterogeneity of plant-pollinator interactions at scales ecologically meaningful to pollinator individuals (but see [12]).

Multiple phenomena can yield small-scale spatial inhomogeneities in plant-pollinator interactions. Firstly, spatial distribution of plants tends to be aggregated at the scales ranging from tens of centimetres to tens of metres [13]. Secondly, foraging ranges of insect pollinators vary from a few hundred metres to a few kilometres [14] and their nest densities may be low (e.g. [15]), contributing heterogeneity in local pollinator distribution. The resulting heterogeneity within sites can translate into differential pollinator visitation and affect both plant and pollinator fitness. For example, reproductive success of individual plants is known to be affected both by neighbourhood floral composition and among-site differences in pollinator composition (e.g. [16], [17], [18], [19]). Similarly, pollinator fecundity and survival can be affected by local environmental heterogeneity [20], [21]. Similarly, spatial differences in plant-pollinator interactions are a factor influencing evolution of floral attraction of generalists versus specialists [22].

The lack of consideration of small-scale spatial structure in plant-pollinator interactions is evident in typical plant-pollinator (especially network) studies, which record plant-pollinator assemblages using sizeable plots (usually with dimensions of several tens of metres). Such approach implicitly assumes spatial homogeneity of plant-pollinator interactions within the plot. This means that the plot-level aggregated pollinator spectrum (recorded species proportions in a pollinator assemblage of a given plant species) represents the pollinator spectrum of each included individual of the species. Moreover, small-scale spatial heterogeneity in plant-pollinator interactions might at least in part underlie the influence that plot size has on the number of interactions discovered per unit of sampling effort (see [23]).

Here, we examine spatial homogeneity of pollinator spectra at a moderately sized mesophytic meadow (largest dimension ca. 260 m) with relatively homogeneous flowering plant composition. It contains minimum obstacles to pollinator movement (presumably allowing pollinators to move according to their preferences). We ask whether plant spatial distribution and consequent variability in small-scale spatial assemblages of plants influence spatial homogeneity of pollinator visitation. In our study, we used a spatially explicit sampling design and quantified both pollinator and flowering plant abundances. Specifically, we ask these questions:

1. Is the pollination network spatially homogeneous at the scale of several tens of meters? I.e. do individuals of the same plant species experience similar pollinator assemblages at different positions within a meadow?
2. How does the local abundance of conspecific plants and highly visited heterospecifics influence the pollinator assemblages of given species?

## Materials and Methods

### Ethics statement

The study did not involve any endangered or protected insect species and complied with the current laws of the Czech Republic. No permissions for this kind of research were necessary.

### Study site

The study was conducted at the K Handrkovu meadow near Vernýřov village, Central Bohemia, Czech Republic (N 49.8466, E 15.1498; WGS 1984). The area of the meadow is 4.5 ha, including unmown verges (ca 0.3 ha). The local climate is moderately sub-oceanic (annual mean temperature around 8°C and annual precipitation around 650 mm; [24]). The vegetation of the meadow could be classified as E3.4 - Moist or wet eutrophic and mesotrophic grassland in the EUNIS classification. There are two peaks of flowering at the meadow (both in terms of diversity and abundance). The first one occurs in May before the first hay cut (beginning of June) and the second one in August before the second hay cut (mid-September).

### Study design

**Figure 1:**
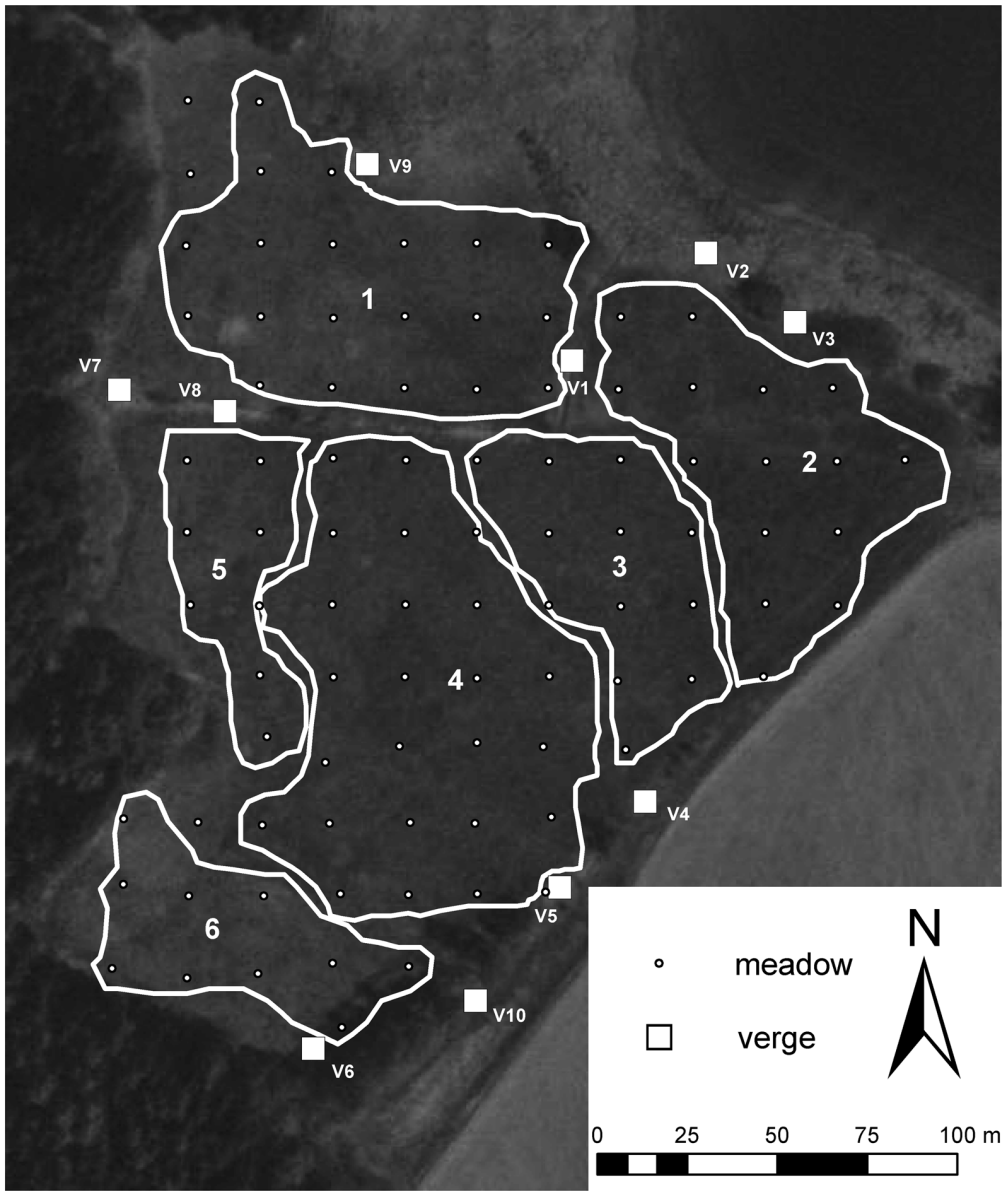
Delimitation of the meadow sectors according to plant community composition. Sectors: 1 – wet, nutrient-poor stands; 2 – mesic to intermittently wet, nutrient-rich stands; 3 – intermittently wet, nutrient moderately rich stands; 4 – mesic, nutrient-rich stands; 5 – moderately wet, nutrient moderately rich stands; 6 – very wet, nutrient moderately rich stand;(for information on flowering plant composition please refer to File S1: Table S4). Aerial photograph credit: Czech Office for Surveying, Mapping and Cadastre.

We used a grid of 93 points spaced 20 m apart, roughly covering the entire meadow, to delineate 93 plots, each centred on one of the grid points (Fig. 1). Each plot measured 4×4 m and was used for censuses of both flowering plants and pollinators. Additionally, 10 plots of size 2×8 m were delineated in the main adjacent linear unmown meadow verges (Fig. 1), since they potentially share insect pollinators with the meadow; these were also used for the censuses. Both plant and pollinator censuses were performed between the 20^th^ and 26^th^ of August 2011, during the second peak flowering period.

Each time a pollinator census was conducted for a given plot, we would record all pollinators visiting insect-pollinated plant species at the time we reached the plot. For the purpose of this study, we assume all flower visitors that were observed to touch the plant's reproductive structures to be pollinators. We are aware that mechanistic evidence for pollination would be necessary to classify the visitors unambiguously. However, most recorded visitors were already found to function as pollinators by other studies. Each pollinator individual was recorded only once, along with the species identity of the visited plant. All meadow plots were censused for pollinators approximately 20 times (range 19–25) and all verge plots approximately 10 times (range 10–11), with observations randomized with respect to date and time of day. Censuses were conducted between 7 and 19 o’clock at weather favourable to insect activity. The pollinators were identified to the lowest taxonomic level possible in the field, after catching the individuals carefully in insect nets. Voucher specimens for morphospecies were collected for Syrphidae and Hymenoptera in order to confirm their identification later. The voucher specimens were deposited at the Dept. of Zoology, Faculty of Science, Charles University in Prague. For the purposes of the presently described study, the observed pollinators were categorized in 12 functional groups: honeybee (*Apis mellifera*), solitary bees, bumblebees (*Bombus* spp.), hoverflies (Syrphidae), true flies (Muscidae), flesh flies (Sarcophagidae), blowflies (Calliphoridae), tachinid flies (Tachinidae), other Diptera, other Hymenoptera, beetles (Coleoptera), and butterflies (Lepidoptera).

A plant census was done once at each plot during the study period. For 17 plant species (chosen based upon previous research at the site showing them to be attractive to hoverflies), the numbers of flowering stalks were counted (see File S1: Tables S1, S2 and Figure S8 for complete list). For the remaining flowering species, abundances were assessed semi-quantitatively by recording the presence/absence of their flowering stalks within a lattice of 64 subplots superimposed over each plot (subplot size 0.5×0.5 m). *Hypericum maculatum* and *H. perforatum* were not distinguished, since they often were interspersed and are indistinguishable without close examination. Here, we report our plant-pollinator interaction results only for those plant species (eight) for which we recorded at least 200 individual visits by pollinators.

Flowering vegetation composition of the plots was summarised by means of detrended correspondence analysis (DCA) in order to identify the main gradients in flowering species composition. We used the sample scores of the plots on the first two ordination axes in further analyses. The first ordination axis explained 14.9% of variability in flowering species composition, corresponding to the moisture gradient (drier towards positive values). The second axis explained 7.7% of variability and could be interpreted as a nutrient or meadow/verge gradient (more nutrients and verge character towards positive values; for details see Figures S1, S2).

For use in addressing Question 1 (degree of spatial homogeneity of pollinator networks), we divided the meadow into several spatially contiguous sectors based upon vegetation similarity (Fig. 1). The delimitation of sectors was done on basis of expert knowledge (Z. Janovský) and took into account all species occurring at the meadow (including grasses and non-flowering species and following the local fine-scale classification [25]). The verge plots were treated individually, with the exception of neighbouring verges nos. 2 and 3, which had very similar vegetation and conditions. In the case of *T. hybridum*, we delimited the sectors at a coarser scale than for other species due to low numbers of visits in the wetter sectors of the meadow, resulting in only two sectors, one in the wetter part of the meadow and the other in the drier part (for details see Figs. S3, S4). When addressing Question 1, the pollinator records for each of the 8 focal plant species were summed across all the plots in each sector.

To address Question 2 (effects of conspecific and heterospecific neighbour abundances), for each of the eight plant species on which we are focusing here, we only used data from plots from which at least five pollinator individuals were recorded for that species. We chose this arbitrary threshold in order to obtain reasonable estimates of pollinator composition (and density) suitable for further analysis. For each focal plant species, due to the varied observation effort at different plots, the pollinator functional group counts were standardized by dividing them by the product of the number of flowering stalks of that species and the number of plot pollinator censuses. Our data therefore represent pollinator functional group densities per flowering stalk and census and we further refer to them as pollinator densities. Densities defined in this way, in contrast to simple per-plot densities, have a straightforward interpretation in terms of potential effects on plant reproduction.

All multivariate analyses were conducted in CANOCO for Windows 4.56 [26].

### Data analysis

For Question 1, differences among the pollinator spectra (i.e. in terms of proportions of individual pollinators accounted for by the pollinator functional groups) of the meadow sectors were evaluated for significance using the χ^2^-test. Data on *S. carvifolia* were not included in this analysis, because it occurred in only one meadow sector. The pollinator groups with low occurrence (i.e. yielding expected values lower than five) were always merged into a category designated as “other” so that the pollinator spectrum matrices met the χ^2^-test assumptions. For *S. officinalis*, this did not suffice to meet the criterion, and therefore for this species the “other” pollinator functional group was not included in the analysis. All computations were done in the R 2.12.0 statistical environment (R Foundation for Statistical Computing, Vienna, http://www.R-project.org/).

For Question 2, the data were analysed by multivariate analyses, namely redundancy analysis (RDA), which is a multivariate extension of multiple regression [27]. Similarly to the analysis for Question 1, rare pollinator groups were placed in the “other” category. For each of the eight plant species for which we had sufficient data, we included the following as predictors: (i) conspecific log-abundances (ii) heterospecific log-abundances of the other focal plant species and (iii) the sample scores along the first two DCA axes for overall flowering plant composition (for details see Figs. S1, S2, for complete lists of predictors [seven to eight per species] considered in forward selection in analyses see File S1: Table S3). Log-abundances of heterospecifics were included for species that occurred in at least 3 of the plots of the given focal species. In the cases of *S. pratensis* and *A. sylvestris*, the plot type (meadow/verge) was also used as a predictor. RDA was used based upon the preliminary DCA analyses, which indicated relative monotony of pollinator functional group responses along the first ordination axis (in all cases gradient length between 1 and 2 S.D.; [28]).

The predictors were tested by means of forward selection and subsequent permutation tests (4999 permutations in each run). In each step, a predictor was tested that explained the most variability. If the first tested predictor was not significant (α<0.05), then the predictor with the second highest explained variability was tested, and so on. If a predictor was significant, we would include it in the model and continue again with testing the predictor with the highest explained variability. The selection ended when no more predictors were found significant.

## Results

**Figure 2:**
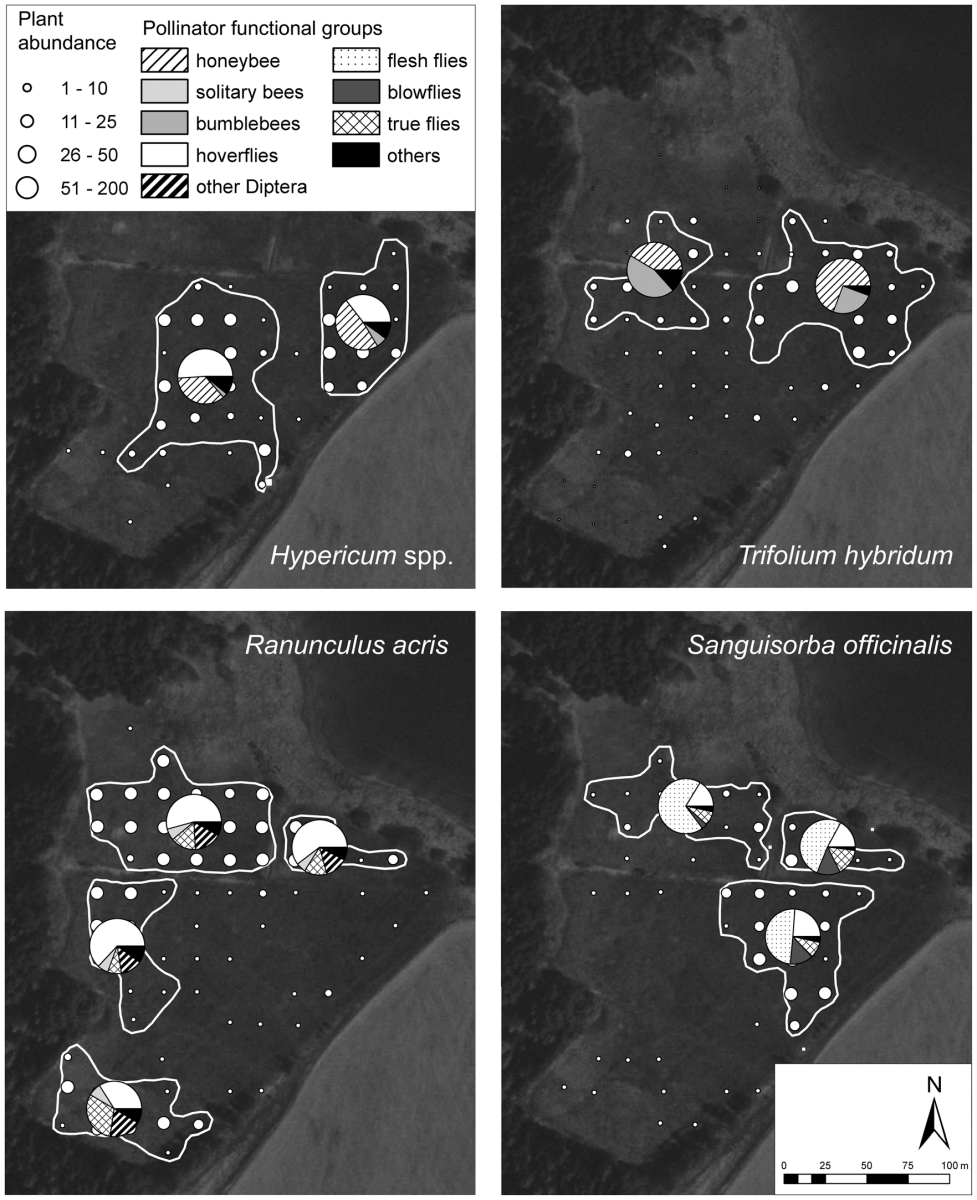
Maps of recorded pollinator spectra for different meadow sectors with more than 50 pollinators recorded for four of the focal plant species. Flowering stalk abundances depicted by size of the dots; please note the different, semi-quantitative scale for *Trifolium hybridum* (0–64 subplots occupied). Please note that in each case the category “others” comprises different pollinator groups (see Materials and Methods for explanation). For more detailed information on pollinator abundances and spectra please refer to Figs. S3, S4. Aerial photograph credit: Czech Office for Surveying, Mapping and Cadastre.

The pollinator spectra of all species included in the test of Question 1 were spatially heterogeneous (Table 1). In the case of *S. officinalis*, one of the functional groups (blow flies – Calliphoridae) was almost completely absent from one of the meadow sectors, constituting a qualitative difference among sectors. For four plant species (*A. sylvestris*, *Hypericum* spp., *R. acris*, and *T. hybridum*), the most abundant pollinator functional group showed substantial differences (16% to 50% change) in the proportions of the pollinator spectrum for which it accounted (Fig. 2 and Fig. S3).

**Table 1:** Summary of occurrence, pollinator spectra, and their differences for the eight most visited plant species; degrees of freedom and P-values are from the χ^2^-tests of homogeneity of pollinator assemblages of the given plant species among different meadow sectors.

plant	no. of occupied plots	no. of flowering stalks	no. of recorded pollinators	main pollinator groups	no. of sectors	df	P-value	main difference
*Angelica sylvestris*	9	100	1281	other Diptera (60%), other Hymenoptera (26%), other (14%)	7	12	<0.001	Varying proportions of other Diptera (29–79%) and other Hymenoptera (13–41%)
*Centaurea jacea*	62	1707	926	honeybee (63%), bumblebees (28%), other (9%)	2	2	0.005	Two-fold difference in bumblebee proportion (15% to 29%)
*Hypericum spp.*	41	1732	291	hoverflies (43%), honeybee (42%), bumblebees (4%), other (11%)	2	3	0.028	Change of dominance between hoverflies and honeybee (51% to 35% and 35% to 48% resp)
*Ranunculus acris*	68	2776	514	hoverflies (52%), true flies (16%), other Diptera (16%), other (16%)	4	9	<0.001	One sector co-dominated by true flies (30%) and hoverflies (32%) instead of hoverflies alone
*Sanguisorba officinalis*	52	888	526	flesh flies (52%), hoverflies (20%), blowflies (13%), true flies (12%), other(3%)	3	6	0.019	Near-absence of blowflies in the wettest sector, higher dominance of flesh flies there (69%)
*Selinum carvifolia*	24	285	355	other Diptera (36%), hoverflies (24%), other Hymenoptera (18%), true flies (10%), other (12%)	1	-	-	-
*Succisa pratensis*	17	203	414	hoverflies (84%), other (16%)	3	2	0.002	Higher proportion of other pollinators in one verge plot (32%)
*Trifolium hybridum*	66	902*	327	honeybee (60%), bumblebees (31%), other (9%)	2	2	<0.001	Bumblebees increase from 25% in the drier sector to 45% in the wetter one

(* Please note that in the case of *T. hybridum*, the no. of flowering stalks corresponds to the number of occupied subplots, for details see Materials and Methods and File S1: Tables S1, S2.)

**Figure 3:**
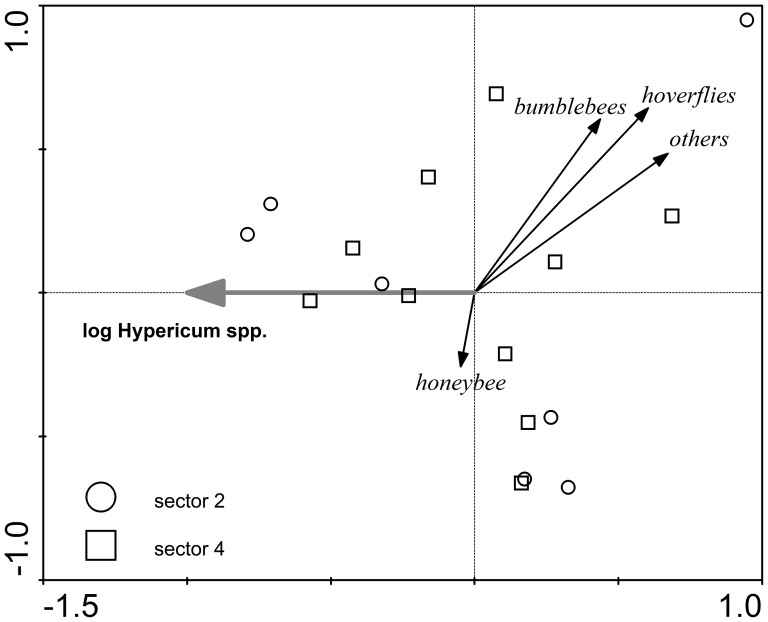
Ordination diagram of RDA of pollinator densities on *Ranunculus acris*. Environmental variables included in the final model, based on forward selection were: logarithm of flowering stalk abundance of *Ranunculus acris* (log R. acris), logarithm of flowering stalk abundance of *Sanguisorba oficinalis* (log S. officinalis), and 1^st^ axis of DCA of flowering plant composition (vegetation 1); plots were categorized according to the sector in which they were located(see Fig. 1 for definition of sectors); 1^st^ ordination axis explains 34.7% of total variability in pollinator density, 2^nd^ axis explains 15.2%.

**Figure 4:**
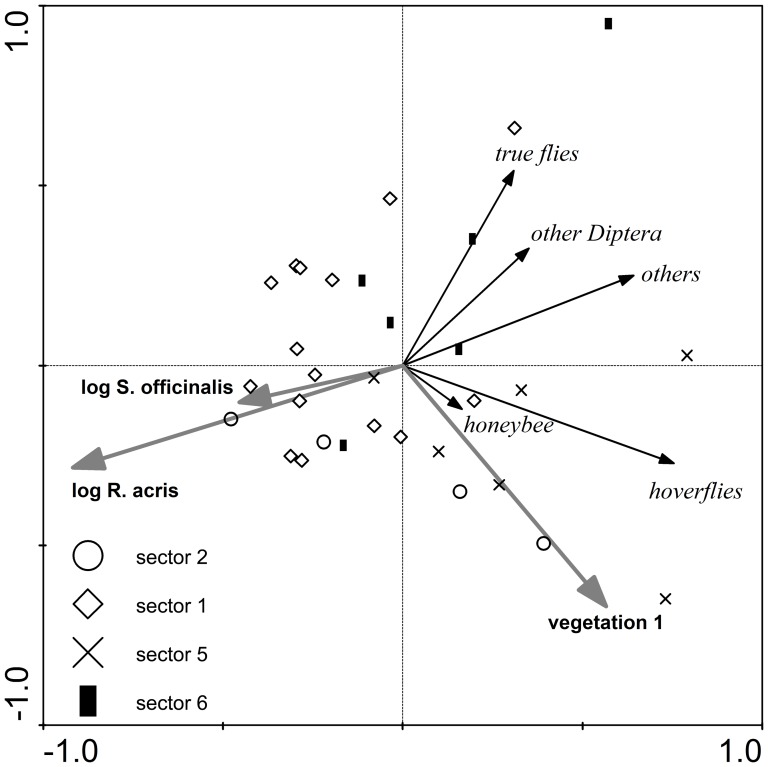
Ordination diagram of RDA analysis of pollinator densities on *Hypericum* spp. Environmental variables included in the final model, based on forward selection were: logarithm of flowering stalk abundance of *Hypericum* spp. (Hypericum (log); plots were categorized according to the sector in which they were located (see Fig. 1 for definition of sectors); 1^st^ ordination axis (canonical) explains 27.5% of total variability in pollinator density, 2^nd^ axis (non-canonical) explains 30.2%.

The multivariate analyses identified significant correlations of pollinator densities with at least one measure of vegetation composition in five of the eight species tested (Table 2). Pollinator spectra of four species (*C. jacea*, *Hypericum* spp., *R. acris*, *S. officinalis*) and marginally one other (*S. carvifolia*) were affected by abundance of conspecifics (Figs. 3 and 4, Fig. S5). Only in the cases of *R. acris* and *T. hybridum* were the pollinator spectra influenced by abundances of other plant species (*S. officinalis* and *R. acris*, respectively).

**Table 2:** Results of the multivariate analyses (RDA with forward selection) of interdependence between plant pollinator densities at each given plot.

plant	no. of plots with >4 pollinators recorded	meadow/verge	DCA of all flowering plants		plant species abundances selected	variability explained
			1^st^ axis	2^nd^ axis		
*Angelica sylvestris*	9	n.s.	n.s.	n.s.	n.s.	
*Centaurea jacea*	30	-	n.s.	n.s.	**Centaurea (0.0064)**; Hypericum (0.0650)	21.9%
*Hypericum* spp.	17	-	n.s.	n.s.	**Hypericum (0.0014)**	27.5%
*Ranunculus acris*	31	-	**0.0048**	n.s.	**Ranunculus (0.0002); Sanguisorba (0.0214)**	49.9%
*Sanguisorba officinalis*	19	-	n.s.	0.0964	**Sanguisorba (0.0008)**	46.0%
*Selinum carvifolia*	10	-	n.s.	n.s.	Selinum (0.0544)	
*Succisa pratensis*	12	n.s.	n.s.	n.s.	n.s.	
*Trifolium hybridum*	17	-	n.s.	n.s.	**Ranunculus (0.0418)**	18.7%

Significant variables (p<0.05) given in bold, marginally significant (p<0.1) given in regular font, “-” denotes variable not included in forward selection (for details see Materials and Methods), variability explained – sum of varibiality in pollinator spectra explained by significant terms

## Discussion

We demonstrate for a relatively large dataset (4634 visits for 8 plant species) that plant-pollinator interactions are spatially heterogeneous at the spatial scale of tens of metres. This was true despite the meadow's moderate size (well within most foraging ranges), isolation (i.e. the detected heterogeneity could not be a reflection of plant or pollinator distributions outside the site) and, importantly, rather homogeneous distribution of all major entomophilous plants over it. In all seven plant species that we had sufficient data to test for the spatial homogeneity of the pollination network, we found different pollinator spectra in different parts of the meadow. The pollinator spectra were influenced both by conspecific densities in the plot and by the densities of other flowering plant species there. The effect of conspecific densities was predominant.

Possible explanations for the observed spatial turnover in plant pollinator interactions include: (i) the interplay between density of a given plant and the per-plant densities of its pollinators; (ii) influence of heterospecific plant densities on pollinators of the constituent plant species (resulting in facilitation or competition); (iii) heterogeneity in pollinator spatial distribution due to abiotic factors or pollinator autecology. Our data can directly address only the first and second possible explanations, but also suggest processes possibly underlying the third explanation.

### The influence of conspecific plant density on pollinator composition and densities

Our results show quite clearly the dependence of the pollinator spectrum of a given plant species on its own density. This was found in four of eight studied species (those with the largest datasets) while in a fifth species, *S. carvifolia*, the same trend was marginally significant. In general, if there was an effect of conspecific density (see Figs. 3 and 4 and Figs. S5, S6, S7) on pollinator abundance, it was negative. For each focal plant, its most abundant pollinator group always decreased with increasing conspecific density. This decrease was usually stronger compared to other pollinator groups. Thus, dominance of the most abundant pollinator group was stronger in plots with low conspecific density, while high conspecific density plots hosted more diverse pollinator spectra. A similar pattern in diversity of pollinator spectra was observed Lázaro et al. [29]. However, they could not attribute it unambiguously to either focal plant density or within-season turnover in plant and pollinator densities (as their data covered the whole flowering season of the species and were pseudoreplicated in time). A possible explanation may be that virtually all available individuals of the dominant pollinator may be attracted to the patch already at lower abundances of the target plant species, while the less common pollinators may be attracted more to the target plant species only at its higher abundances.

Further, the overall increase in pollinator abundances did not match the increase of target plant abundances, leading to decreases in pollinator densities and possible increases in intra-specific competition for pollinators. The results of previous studies have been mixed, with some reporting positive associations between target plant densities and pollinator visitation rates [16], [30] and some finding no association [31], [32] or even a negative association [33]. Since many of the studies reporting positive effects did not use a visitation rate standardized per individual plant, and the slopes of their visitation plotted against plant density were often lower than one, we believe that the occurrence of positive effects has been overstated. Thus, we suggest that pollinator saturation may be a commoner phenomenon than previously thought.

Nonetheless, it is less clear whether the observed decrease of pollinator densities and increase of diversity of pollinator spectra in high conspecific density plots effectively translates into decrease in plant reproductive output per unit reproductive effort. Indeed, studies showing no or positive effect of conspecific density on fitness prevail [16], [17], [34], [35]. These outcomes could be explained either by the fact that even the recorded “low” pollinator densities did not cause pollen limitation, or other properties of high conspecific density stands outweighed the negative effects of lower pollinator densities. On the other hand, in systems including pollinators of very different effectiveness (carryover capacity; sensu [19]), differences in pollinator spectrum composition probably translate into differences in reproductive success [18]. This is not the case for most of our eight focal species, since the three most common pollinator groups in our system– honeybee, bumblebees, and hoverflies – have similar effectiveness [36]. However, it might play a role for *R. acris* and *S. officinalis*, which are visited both by furry dipterans (most hoverflies) and non-furry dipterans, which are reported to have much lower carryover capacity (i.e. effectiveness) [19].

### The influence of abundances of heterospecific flowering plants

The effects of heterospecific densities on pollinator spectra were detected only in two species (*R. acris* and *T. hybridum*). In the case of *R. acris*, the density of a neighbour species, *S. officinalis*, affected the pollinator spectrum in the same way as conspecific density, i.e. decreased per flower stalk densities of all pollinator groups. Hoverflies and true flies, the key pollinators of *R. acris*, also visit *S. officinalis*, but the relationship between these two plants and their pollinators is asymmetric in that flesh flies (the main pollinators of *S. officinalis*) scarcely visit *R. acris*. This contrasts with the predominantly positive interspecific interactions among plant species found by Hegland et al. [37] in a similar system in southern Norway. This difference in findings may have been caused by different overall flower densities, with negative interactions starting to outweigh the interspecific facilitation only at high floral densities (e.g. [38]). Mutually negative relationships have also been reported from systems involving closely related species with similar floral displays (e.g. [17], [39]), which, however, was not the case here.

The moisture gradient in overall floral composition (1^st^ axis of vegetation DCA) was correlated only with hoverfly abundances on *R. acris* (positively towards drier areas). This outcome may reflect two trends in our data: (i) decreasing overall floral dominance of *R. acris* and (ii) the presence of most other hoverfly-sharing plant species only in wetter parts of the meadow. Unlike in wetter parts, the generalist pollinators not preferring *R. acris* would not need to visit it in drier parts of the meadow with abundant preferred plant species. On the other hand, hoverflies visiting *R. acris* have to concentrate on it in drier meadow parts where they lack alternative visited species (with the exception of *S. officinalis*).

We suggest that the effect of *R. acris* densities on pollinators of *Trifolium hybridum* is an artefact, with *R. acris* only being better than the 1^st^ DCA axis as a surrogate for moisture gradient. In general, we suggest that the test power of our relatively large dataset is still quite low for revealing effects of heterospecific plant abundances (unlike for conspecific abundances).

### Other possible causes of heterogeneity in pollinator spatial distributions

Although we assume, based upon the pollinator foraging distances, that pollinators can reach all their preferred plots and plant species within our study meadow, it is uncertain whether they really do. Optimal foraging theory [40] predicts that preference for a certain host plant should be a combination of its profitability (e.g. net of energy gain from nectar) and its distance (i.e. decrease in encounter rate). Thus, despite the presence of preferred sources, the proportion of pollinators visiting suboptimal but nearer sources should increase with increasing mismatch between the breeding/emerging sites of pollinators and the locations of their preferred plant sources. This could be especially true in the case of Hymenoptera, which must return repeatedly to their nests. Additionally, various phenomena could cause pollinators to avoid foraging in some areas, e.g. for bumblebees, the immediate vicinity of their nest [15], [41]. All these factors are likely to influence encounter probabilities and mobility, the key factors structuring mutualist networks (see [11]). Direct competition among pollinators (e.g. [42], [43]) could also influence spatial distribution of pollinator densities. If floral resources were limiting, we would not observe a relationship between conspecific plant densities and pollinator densities per plant, because the floral resource would be saturated with pollinators. Since we observed a decrease in pollinator densities per plant with increasing conspecific plant densities, direct competition does not seem to affect considerably our system.

Abiotic factors, particularly shading, might influence pollinator spatial distribution in our system. The bordering forest shades some of the plots, yielding differences in light period of up to three hours. Most pollinators ceded to visit shaded plots, but bumblebees continued to visit them, it might be due to their larger size and partial thermoregulation [15]. Plot wetness could also affect the pollinator spectra, with rising proportions of true flies and flesh flies in the spectra of *R. acris* and *S. officinalis* in wetter areas (possibly due to nearness of emergence sites).

### Implications for interpreting plant-pollinator interactions

Our results indicating strong spatial heterogeneity of plant-pollinator interactions have two main implications, each explored below: (i) plot size and sampling effort allocation in plant-pollinator studies needs to take this heterogeneity into account; (ii) spatial structure of sampling effort may potentially change the probability of detection of modules in pollination networks [sensu 7] (both probabilities of false negatives and false positives).

We found significant spatial effects on pollinator spectra at the scales of tens of metres, which suggests that results obtained by averaging data from large plots or transects (commonly measuring even 100 metres) do not provide a reliable representation of pollinator spectra experienced by individual plants. Sampling plot heterogeneity increases the probability of discovering a particular plant-pollinator interaction and thus decreases the probability of falsely designating species as specialists (see [44]). However, it can also create what we would term “false generalists”, since the term generalist may both apply to a species, where also the individuals behave as generalists, or to a “false generalist” species, whose individuals actually visit narrower but differing spectra of plants (or they even act as specialists). While there is no difference among such species from viewpoint of pollinator ecology, the plants perceive the second species as more specialised with corresponding benefits for pollination. Large heterogeneous plots increase probability of including areas, where the pollinator individuals actually visit narrower plant spectra.

Moreover, the cumulative pollinator spectra (i.e. from all individuals of a pollinator across an entire study site) recorded will be influenced by the degree to which the spatial distribution of sampling corresponds to the heterogeneity of these interactions. Gibson et al. [23] recommended even sampling effort allocation with respect to overall plant abundances. Based upon our results, we suggest extending this recommendation to even sampling of the whole range of conspecific plant densities at the site, or better yet to divide the site into subplots of pollinator-meaningful size and then sample them evenly. It is an open question how small-scale spatial heterogeneity in plant-pollinator interactions translates into higher order pollination network properties. However, increased spatial heterogeneity in plant-pollinator interactions requires greater sampling to reliably describe the plant-pollinator interactions. This makes more pronounced the common problem of undersampling of pollinator networks (cf. [45]) known to affect higher order network properties [46], [47].

Modules in plant-pollinator networks have been proposed as possible co-evolutionary units, where selection could act on both plants and pollinators [7]. This would require the modules to be stable both in space and time and relatively isolated in terms of gene flow. Moreover, the observed spatial heterogeneity in pollinator visitation can possibly have significant effects on probabilities of delimitation of network modules, depending on the sampled part of the meadow. For example, blow flies (Calliphoridae) were, in one of our meadow sectors, at most only an accessory pollinator group of *S. officinalis*, whereas in the rest of the meadow they were the third most common visitors to this species, only rarely visiting other plants. Thus, the chances of delimiting a pollination-network module around *S. officinalis* differed greatly over the scale of only tens of metres.

Taken together, our results imply that the influences of local context (i.e. conspecific and heterospecific flowering plant densities) are not only detectable in plant-pollinator networks, but also exert relatively strong influence on their structure. Therefore the plant-pollinator networks should consider more the spatial aspect of their sampling structure.

## Supporting Information

Figure S1 **Ordination diagram of species centroids for DCA of flowering species composition.** first and second axis depicted with 14.9% and 7.7% of variability explained respectively; only species with weight greater than 2% shown; for explanation of abbreviations, see Tables S1 and S2. Altogether 57 flowering plant species were recorded within 103 plots. The first axis explained 14.9% of variability in lowering plant species composition; the second axis explained 7.7% of variation. Downweighting of rare species was applied. The length of the gradient of the first axis was 5.441 suggesting the selected unimodal technique was an appropriate choice. The depicted axes could be interpreted as wetness and nutrient or meadow/verge gradient respectively.
(PNG)

Figure S2 **Ordination diagram for species centroids (triangle) and sample scores for DCA of flowering species composition.** first and second axis depicted with 14.9% and 7.7% of variability explained respectively; white squares denote meadow plots and grey ones verge plots; only species with weight greater than 2% shown; for explanation of abbreviations, see Tables S1 and S2.
(PNG)

Figure S3 **Maps of delimited sectors for individual plant species under study and their pollinator assemblages.** numbers next to pies indicate number of pollinators the pie is based on. Others denotes always all remaining distinguished pollinator functional groups, which do not have a separate field; abundance of focal plant species depicted on background, (for complete legend please refer Fig. S8); A) *A. sylvestris*; B) *C. jacea*; C) *Hypericum* spp.; D) *R. acris*; E) *S. officinalis*; F) *S. pratensis*; G) *T. hybridum*; Please note that only one sector was delimited in *S. carvifolia* and therefore it was not included into analysis of pollinator assemblages according to sectors. Aerial photograph credit: Czech Office for Surveying, Mapping and Cadastre.
(TIF)

Figure S4 **Maps of pollinator assemblages at individual plots with more than five recorded pollinators at the givern focal species.** numbers next to pies indicate number of pollinators the pie is based on. Abundance of focal plant species depicted on background, (for complete legend please see Fig. S8); A) *A. sylvestris*; B) *C. jacea*; C) *Hypericum* spp.; D) *R. acris*; E) *S. officinalis*; F) *S. carvifolia*; G) *S. pratensis*; H) *T. hybridum*. Aerial photograph credit: Czech Office for Surveying, Mapping and Cadastre.
(TIF)

Figure S5 **Ordination diagram of RDA analysis of pollinator densities on** ***Centaurea jacea*** **, forward selection has identified as environmental variables included into the final model only logarithm of flowering stalk abundance of** ***C. jacea*** **(log C. jacea).** plots are categorized according to the sector of origin (see Fig. 1 for definition of sectors); 1^st^ ordination axis explains 21.9% of total variability in pollinator density, 2^nd^ axis explains 66.0%.
(PNG)

Figure S6 **Ordination diagram of RDA analysis of pollinator densities on** ***Sanguisorba officinalis*** **.** Forward selection has identified as environmental variables included into the final model only logarithm of flowering stalk abundance of *S. officinalis* (log S. officinalis); plots are categorized according to the sector of origin (see Fig. 1 for definition of sectors); 1^st^ ordination axis explains 46.0% of total variability in pollinator density, 2^nd^ axis explains 44.4%.
(PNG)

Figure S7 **Ordination diagram of RDA analysis of pollinator densities on** ***Trifolium hybridum*** **, forward selection has identified as environmental variables included into the final model only logarithm of flowering stalk abundance of** ***Ranunculus acris*** **(log R. acris).** plots are categorized according to the sector of origin (see Fig. 1 for definition of sectors); 1^st^ ordination axis explains 18.7% of total variability in pollinator density, 2^nd^ axis explains 54.4%.
(PNG)

Figure S8 **Maps of occurrence and abundance of the eight focal plant species pollinator assemblages at individual plots.** the symbol sizes indicate abundance categories as noted in legend; light green symbols stand for meadow plots and light blue for verge plots; please note the different scale in *T. hybridum* referring to the number of subplots occupied instead of number of flowering stalks; A) *A. sylvestris*; B) *C. jacea*; C) *Hypericum* spp.; D) *R. acris*; E) *S. officinalis*; F) *S. carvifolia*; G) *S. pratensis*; H) *T. hybridum*. Aerial photograph credit: Czech Office for Surveying, Mapping and Cadastre.
(TIF)

File S1 **File containing Tables S1–S4.**
(DOC)
