# Supplementary figures and images for: Conspecific and Heterospecific Plant Densities at Small-Scale Can Drive Plant-Pollinator Interactions
Source: PLoS One. 2013 Oct 21;8(10):e77361. doi: 10.1371/journal.pone.0077361 (PMC3804547; doi:10.1371/journal.pone.0077361)

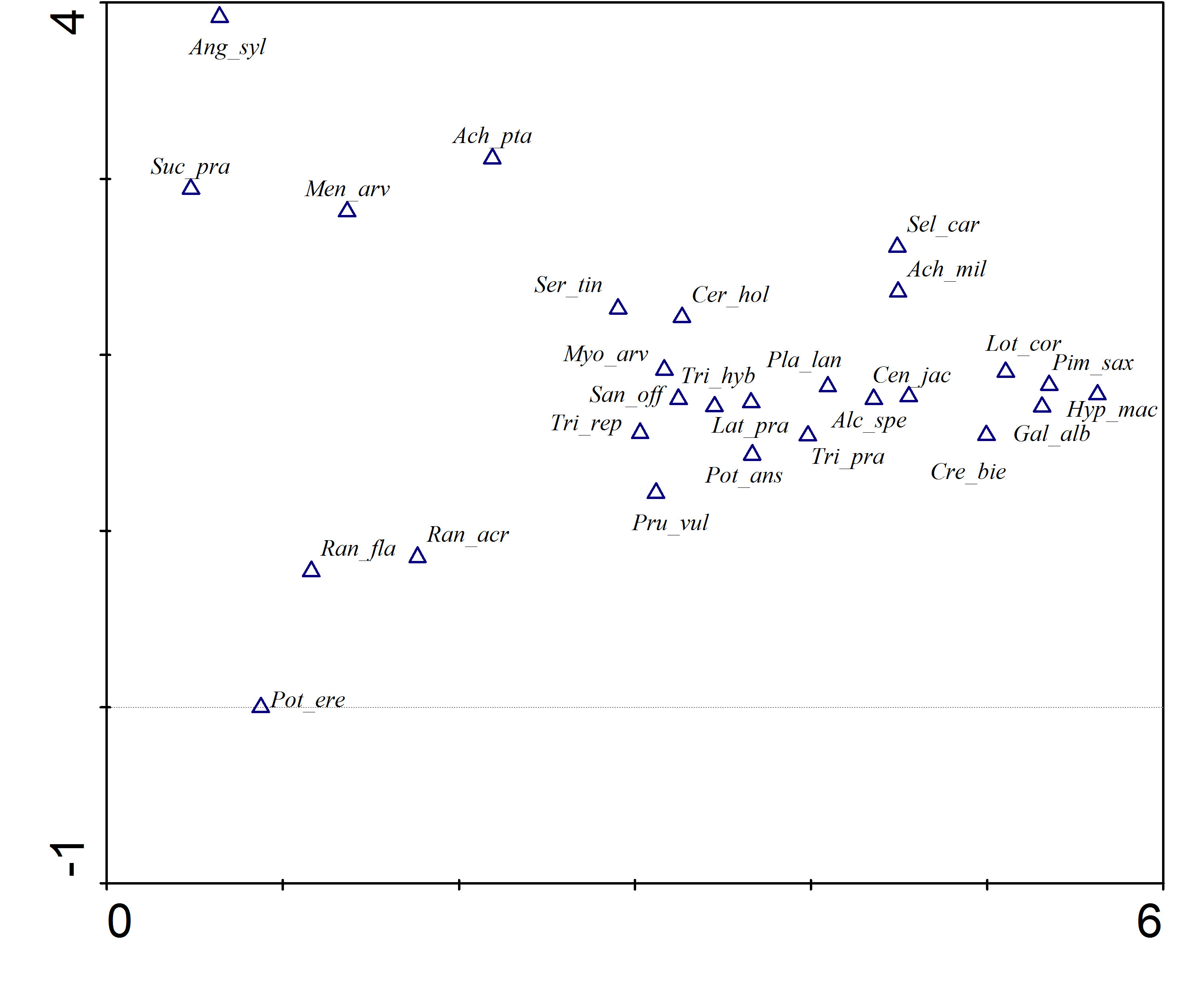

Supplement: Figure S1 — Ordination diagram of species centroids for DCA of flowering species composition. first and second axis depicted with 14.9% and 7.7% of variability explained respectively; only species with weight greater than 2% shown; for explanation of abbreviations, see Tables S1 and S2. Altogether 57 flowering plant species were recorded within 103 plots. The first axis explained 14.9% of variability in lowering plant species composition; the second axis explained 7.7% of variation. Downweighting of rare species was applied. The length of the gradient of the first axis was 5.441 suggesting the selected unimodal technique was an appropriate choice. The depicted axes could be interpreted as wetness and nutrient or meadow/verge gradient respectively. (PNG) [file pone.0077361.s001.png]

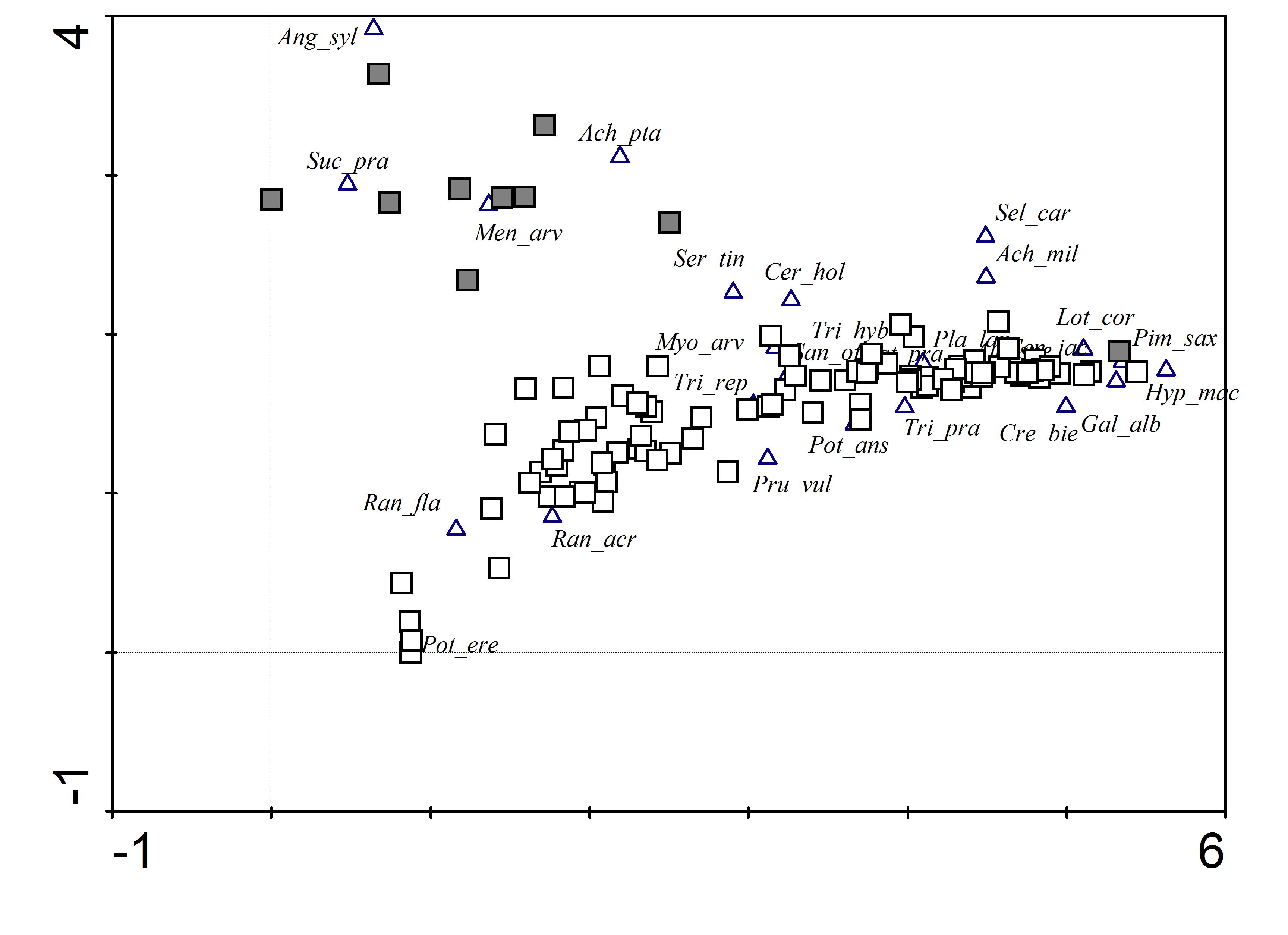

Supplement: Figure S2 — Ordination diagram for species centroids (triangle) and sample scores for DCA of flowering species composition. first and second axis depicted with 14.9% and 7.7% of variability explained respectively; white squares denote meadow plots and grey ones verge plots; only species with weight greater than 2% shown; for explanation of abbreviations, see Tables S1 and S2. (PNG) [file pone.0077361.s002.png]

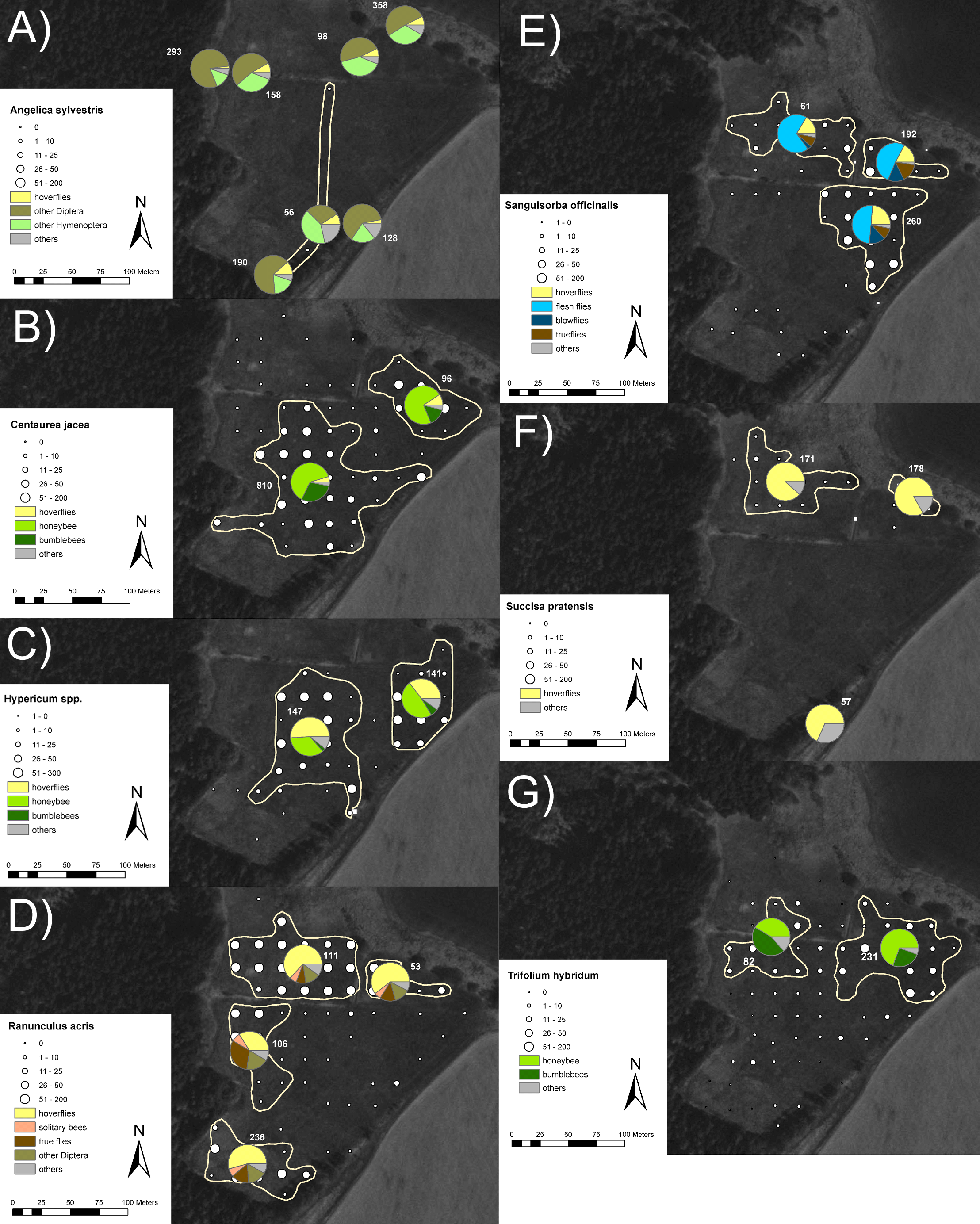

Supplement: Figure S3 — Maps of delimited sectors for individual plant species under study and their pollinator assemblages. numbers next to pies indicate number of pollinators the pie is based on. Others denotes always all remaining distinguished pollinator functional groups, which do not have a separate field; abundance of focal plant species depicted on background, (for complete legend please refer Fig. S8); A) A. sylvestris; B) C. jacea; C) Hypericum spp.; D) R. acris; E) S. officinalis; F) S. pratensis; G) T. hybridum; Please note that only one sector was delimited in S. carvifolia and therefore it was not included into analysis of pollinator assemblages according to sectors. Aerial photograph credit: Czech Office for Surveying, Mapping and Cadastre. (TIF) [file pone.0077361.s003.tif]

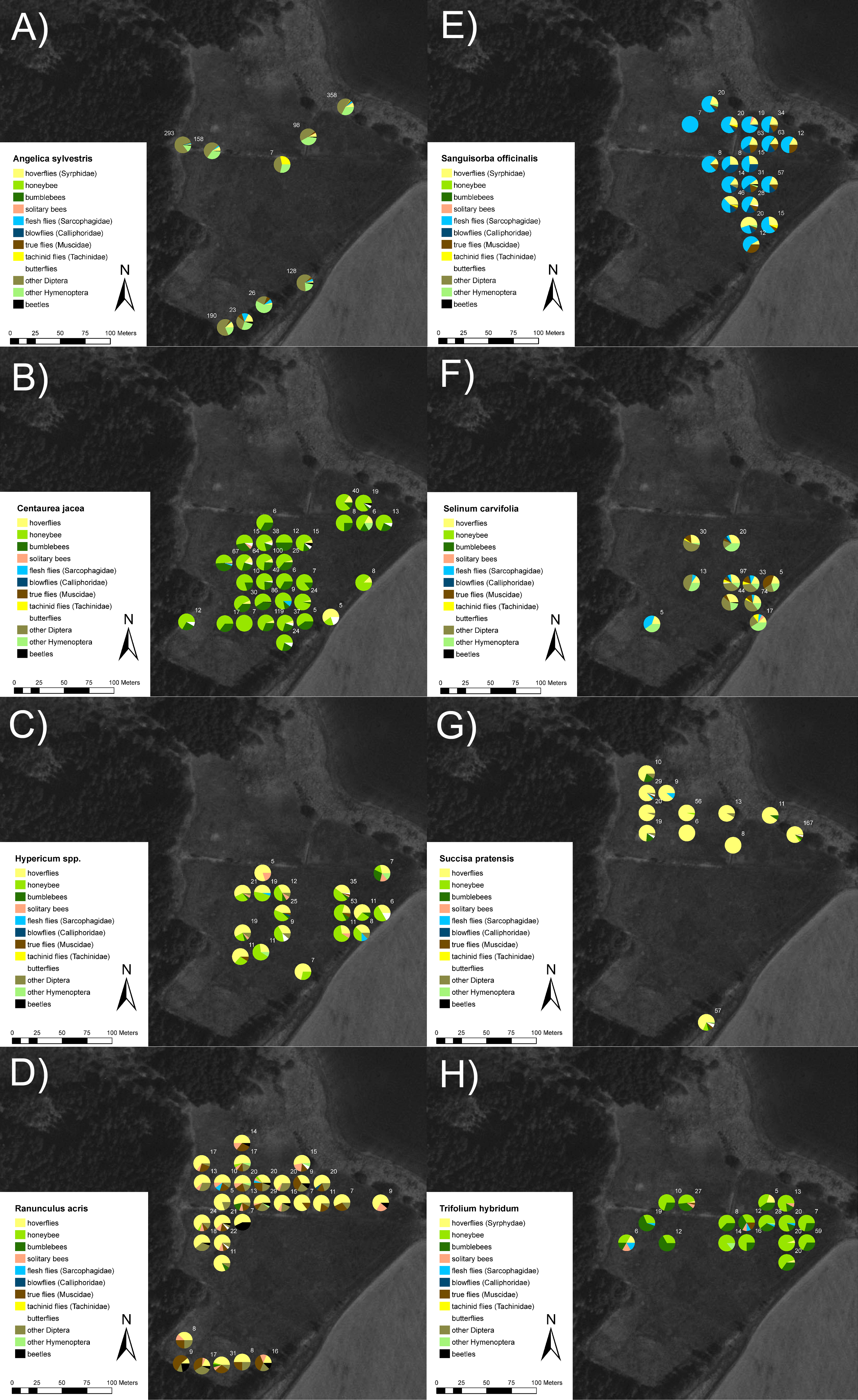

Supplement: Figure S4 — Maps of pollinator assemblages at individual plots with more than five recorded pollinators at the givern focal species. numbers next to pies indicate number of pollinators the pie is based on. Abundance of focal plant species depicted on background, (for complete legend please see Fig. S8); A) A. sylvestris; B) C. jacea; C) Hypericum spp.; D) R. acris; E) S. officinalis; F) S. carvifolia; G) S. pratensis; H) T. hybridum. Aerial photograph credit: Czech Office for Surveying, Mapping and Cadastre. (TIF) [file pone.0077361.s004.tif]

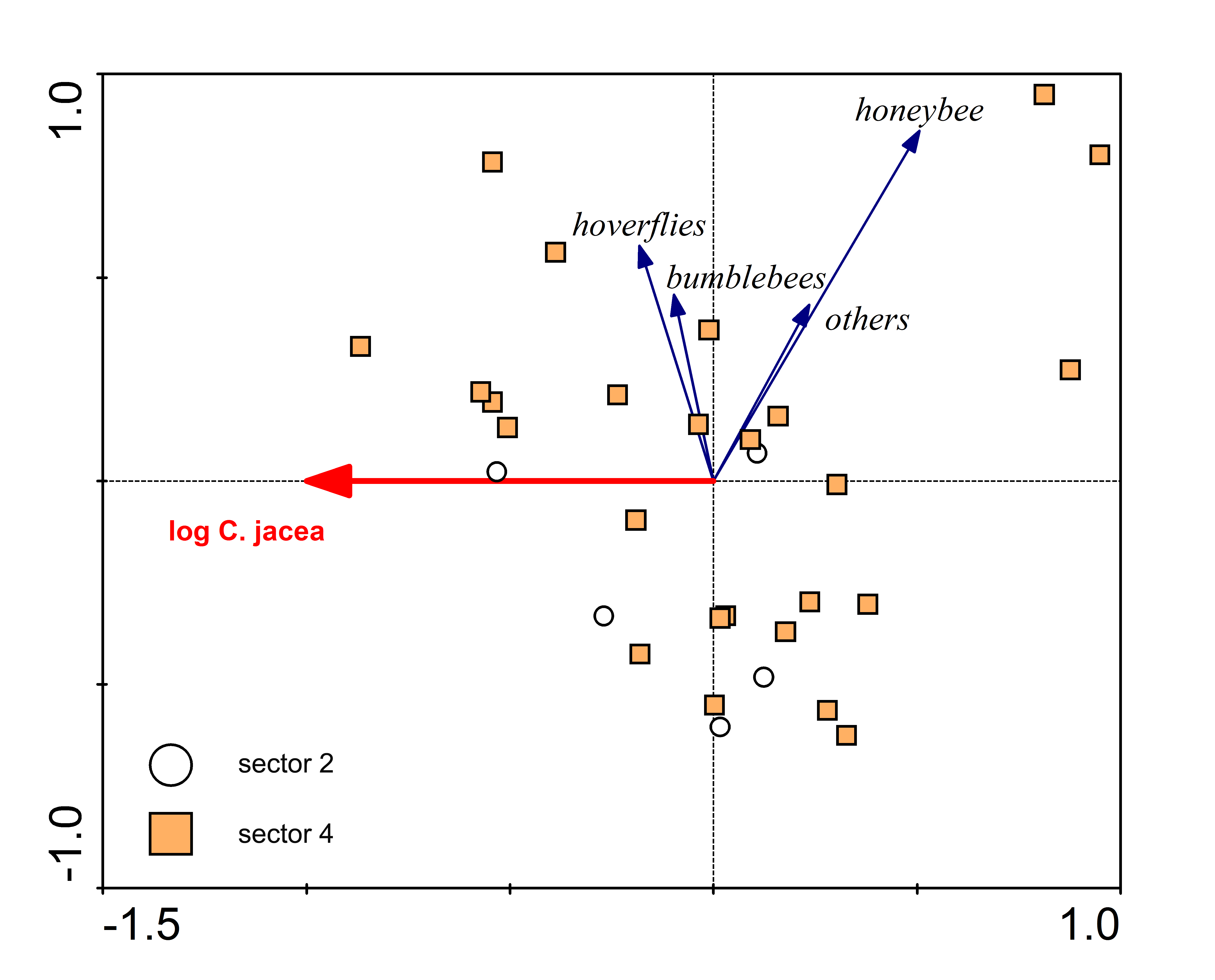

Supplement: Figure S5 — Ordination diagram of RDA analysis of pollinator densities on Centaurea jacea , forward selection has identified as environmental variables included into the final model only logarithm of flowering stalk abundance of C. jacea (log C. jacea). plots are categorized according to the sector of origin (see Fig. 1 for definition of sectors); 1st ordination axis explains 21.9% of total variability in pollinator density, 2nd axis explains 66.0%. (PNG) [file pone.0077361.s005.png]

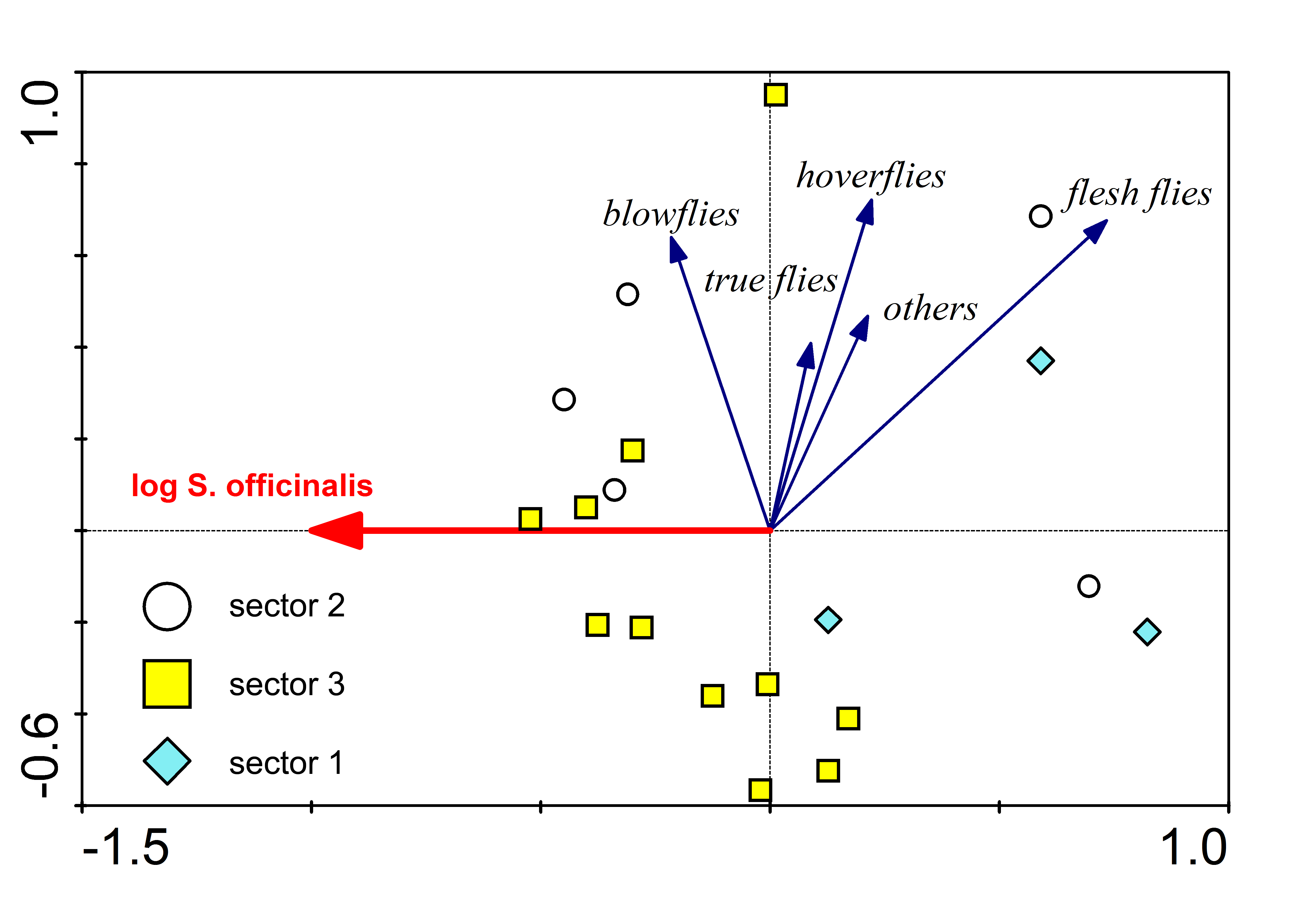

Supplement: Figure S6 — Ordination diagram of RDA analysis of pollinator densities on Sanguisorba officinalis . Forward selection has identified as environmental variables included into the final model only logarithm of flowering stalk abundance of S. officinalis (log S. officinalis); plots are categorized according to the sector of origin (see Fig. 1 for definition of sectors); 1st ordination axis explains 46.0% of total variability in pollinator density, 2nd axis explains 44.4%. (PNG) [file pone.0077361.s006.png]

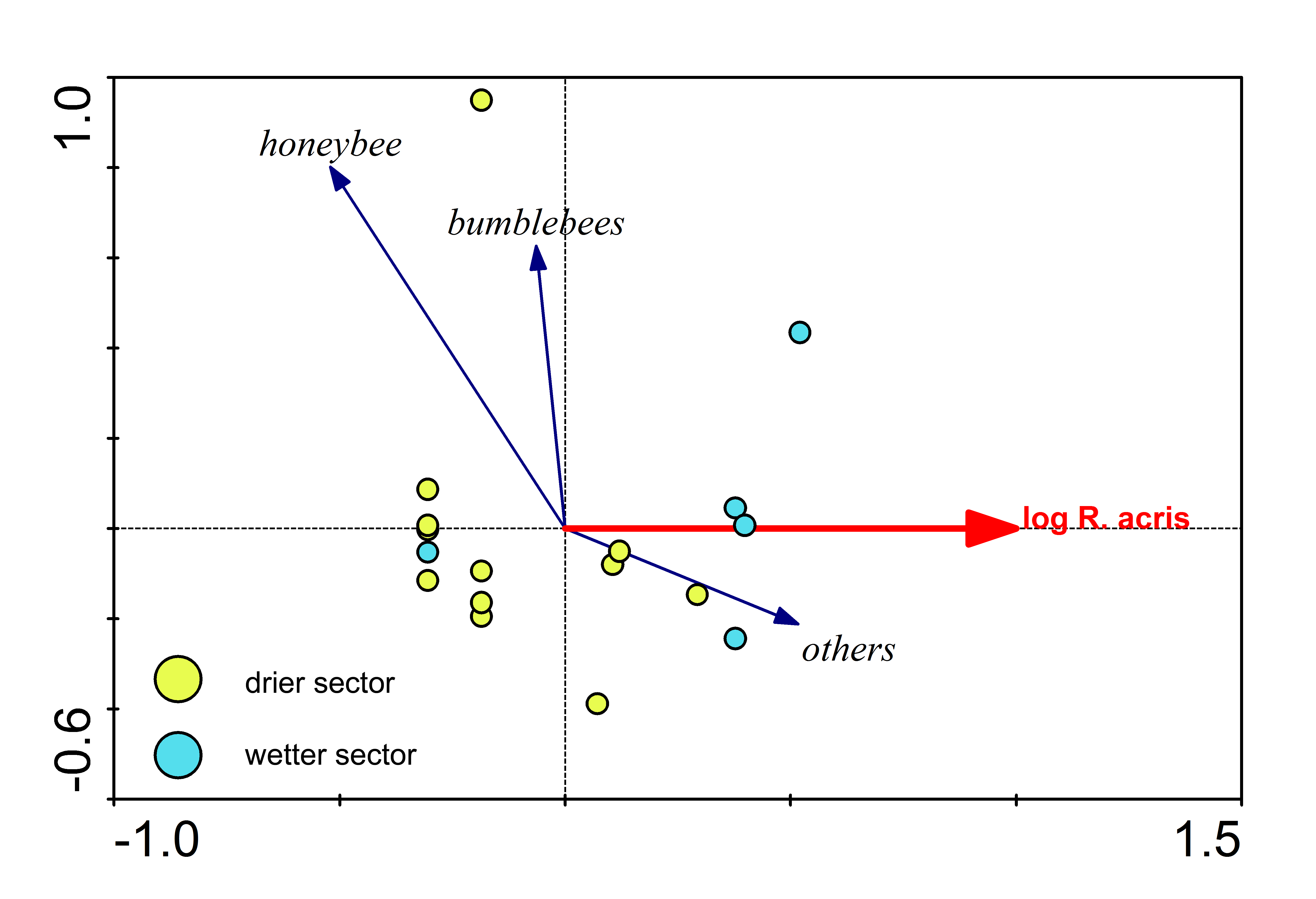

Supplement: Figure S7 — Ordination diagram of RDA analysis of pollinator densities on Trifolium hybridum , forward selection has identified as environmental variables included into the final model only logarithm of flowering stalk abundance of Ranunculus acris (log R. acris). plots are categorized according to the sector of origin (see Fig. 1 for definition of sectors); 1st ordination axis explains 18.7% of total variability in pollinator density, 2nd axis explains 54.4%. (PNG) [file pone.0077361.s007.png]

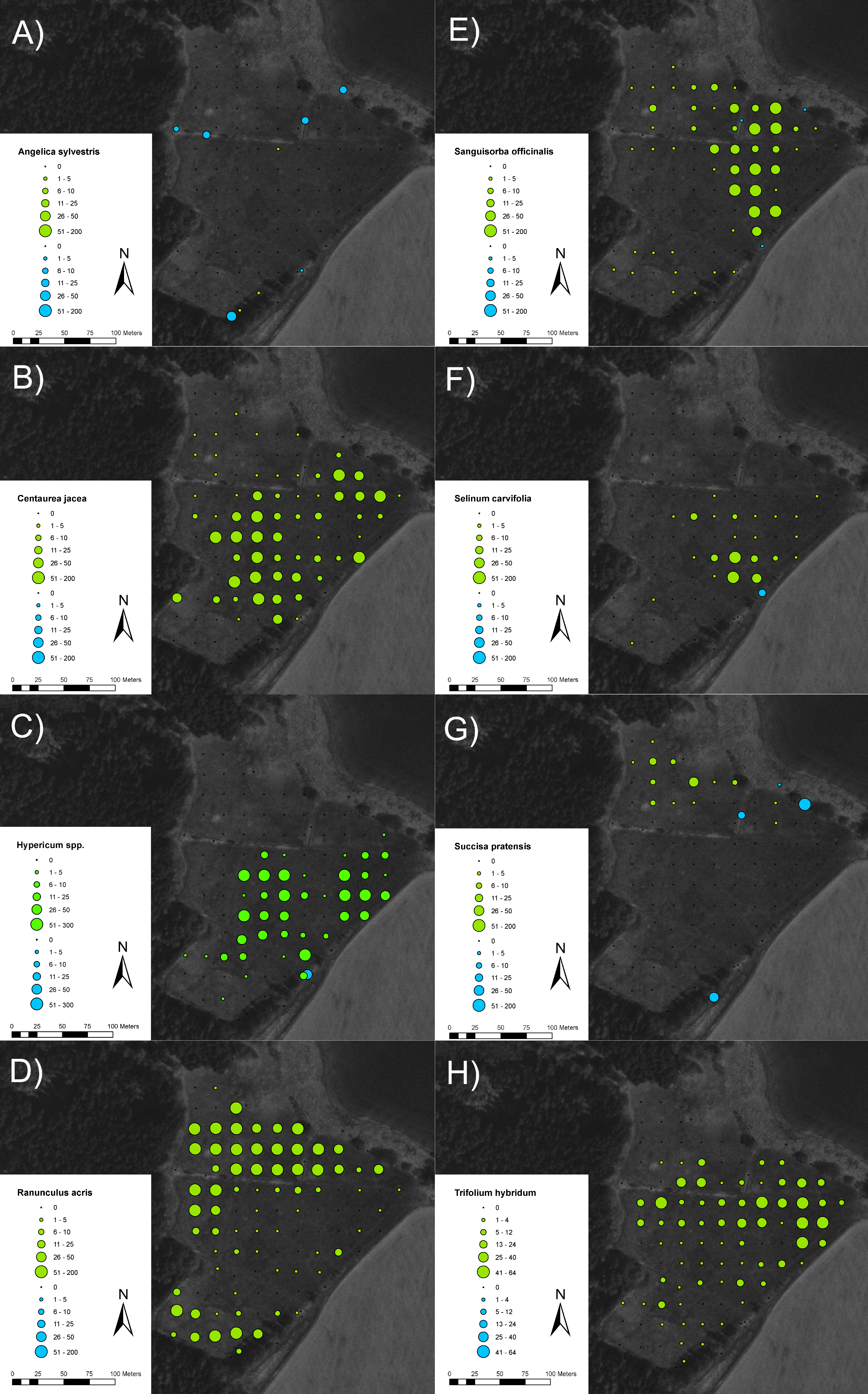

Supplement: Figure S8 — Maps of occurrence and abundance of the eight focal plant species pollinator assemblages at individual plots. the symbol sizes indicate abundance categories as noted in legend; light green symbols stand for meadow plots and light blue for verge plots; please note the different scale in T. hybridum referring to the number of subplots occupied instead of number of flowering stalks; A) A. sylvestris; B) C. jacea; C) Hypericum spp.; D) R. acris; E) S. officinalis; F) S. carvifolia; G) S. pratensis; H) T. hybridum. Aerial photograph credit: Czech Office for Surveying, Mapping and Cadastre. (TIF) [file pone.0077361.s008.tif]
