# Supplementary material for: Conspecific and Heterospecific Plant Densities at Small-Scale Can Drive Plant-Pollinator Interactions
Source: PLoS One. 2013 Oct 21;8(10):e77361. doi: 10.1371/journal.pone.0077361 (PMC3804547; doi:10.1371/journal.pone.0077361)
Supplement: File S1 — File containing Tables S1–S4. (DOC) [file pone.0077361.s009.doc]

Table S1 - Abundances and numbers of recorded pollinators for plants species recorded by direct counting of the flowering stalks; species given in bold were included in analyses

| plant name | plant abbr. | no. of occupied plots | no. of flowering stalks | no. of recorded pollinators |
| --- | --- | --- | --- | --- |
| **Ranunculus acris** | **Ran_acr** | **68** | **2776** | **514** |
| **Centaurea jacea** | **Cen_jac** | **62** | **1707** | **926** |
| Achillea ptarmica | Ach_pta | 56 | 847 | 111 |
| **Sanguisorba officinalis** | **San_off** | **52** | **888** | **526** |
| **Hypericum spp.*** | **Hyp_spp** | **41** | **1732** | **291** |
| Pimpinella saxifraga | Pim_sax | 35 | 379 | 156 |
| Crepis biennis | Cre_bie | 24 | 318 | 76 |
| **Selinum carvifolia** | **Sel_car** | **24** | **285** | **355** |
| **Succisa pratensis** | **Suc_pra** | **17** | **203** | **414** |
| Serratula tinctoria | Ser_tin | 16 | 96 | 25 |
| Achillea millefolium agg. | Ach_mil | 13 | 113 | 69 |
| Leucanthemum vulgare agg. | Leu_vul | 12 | 28 | 25 |
| Cirsium palustre | Cir_pal | 10 | 19 | 13 |
| **Angelica sylvestris** | **Ang_syl** | **9** | **100** | **1281** |
| Daucus carota | Dau_car | 9 | 29 | 134 |
| Heracleum sphondylium | Her_sph | 6 | 14 | 166 |
| Cirsium arvense | Cir_arv | 5 | 13 | 9 |
| Lythrum salicaria | Lyt_sal | 4 | 57 | 34 |

* *Hypericum maculatum* and *H. perforatum* are reported here together, since they were not distinguished during the pollinator census. *H. maculatum* is much more common with 37 occupied plots and 1710 flowering stalks compared to *H. perforatum* with 9 occupied plots and 22 flowering stalks.

Table S2 - Abundances and numbers of recorded pollinators for plants species recorded semi-quantitatively by direct counting of the flowering stalks; species given in bold were included in analyses

| plant name | plant abbr. | no. of occupied plots | no. of occupied subplots | no. of recorded pollinators |
| --- | --- | --- | --- | --- |
| *Trifolium repens* | Tri_rep | 72 | 1003 | 132 |
| ***Trifolium hybridum*** | **Tri_hyb** | **66** | **902** | **327** |
| *Plantago lanceolata* | Pla_lan | 63 | 1380 | 148 |
| *Lathyrus pratensis* | Lat_pra | 51 | 489 | 39 |
| *Prunella vulgaris* | Pru_vul | 51 | 368 | 21 |
| *Lotus corniculatus* | Lot_cor | 45 | 819 | 21 |
| *Galium album* | Gal_alb | 43 | 695 | 13 |
| *Potentilla erecta* | Pot_ere | 41 | 582 | 16 |
| *Alchemilla* sp. | Alc_spe | 40 | 159 | 0 |
| *Trifolium pratense* | Tri_pra | 34 | 256 | 75 |
| *Mentha arvensis* | Men_arv | 27 | 152 | 7 |
| *Ranunculus flammula* | Ran_fla | 26 | 250 | 36 |
| *Cerstium holosteoides* | Cer_hol | 20 | 57 | 0 |
| *Myosotis* sp. | Myo_arv | 18 | 61 | 3 |
| *Vicia tetrasperma* | Vic_tet | 16 | 48 | 0 |
| *Galium uliginosum* | Gal_uli | 15 | 36 | 0 |
| *Potentilla anserina* | Pot_ans | 10 | 64 | 5 |
| *Polygonum* sp. | Pol_spe | 7 | 21 | 0 |
| *Epilobium* sp. | Epi_spe | 7 | 9 | 0 |
| *Campanula patula* | Cam_pat | 7 | 7 | 0 |
| *Lysimachia vulgaris* | Lys_vul | 4 | 45 | 0 |
| *Anagallis arvensis* | Ana_arv | 4 | 20 | 0 |
| *Lysimachia nummularia* | Lys_num | 4 | 9 | 0 |
| *Stellaria graminea* | Ste_gra | 4 | 4 | 0 |
| *Leontodon hispidus* | Leo_his | 3 | 44 | 34 |
| *Gnaphalium sylvaticum* | Gna_syl | 3 | 5 | 0 |
| *Scutellaria galericulata* | Scu_gal | 2 | 10 | 0 |
| *Trifolium dubium* | Tri_dub | 2 | 4 | 0 |
| *Leontodon autumnalis* | Leo_aut | 2 | 2 | 0 |
| *Vicia hirta* | Vic_hir | 1 | 6 | 0 |
| *Lycopus europaeus* | Lyc_eur | 1 | 5 | 0 |
| *Odontites vernus* | Odo_ver | 1 | 2 | 0 |
| *Vicia cracca* | Vic_cra | 1 | 2 | 0 |
| *Vicia sepium* | Vic_sep | 1 | 2 | 0 |
| *Cirisium canescens* | Cir_can | 1 | 1 | 18 |
| *Lychnis flos-cuculi* | Lyc_flo | 1 | 1 | 0 |
| *Medicago lupulina* | Med_lup | 1 | 1 | 0 |
| *Polygonum aviculare* | Pol_avi | 1 | 1 | 0 |

Table S3 – Factors included in forward selection of variables in RDA analyses of pollinator composition on focal species. Abbreviations: A. syl. – *Angelica sylvestris*, C. jac. – *Centaurea jacea*, H. spp. – *Hypericum* spp., R. acr. – *Ranunculus acris*, S. off. – *Sanguisorba officinalis*, S. car. – *Selinum carvifolia*, S. pra. – *Succisa pratensis*, T. hyb. – *Trifolium hybridum*

| factors included in forw. selection | A. syl. | C. jac. | H. spp. | R. acr. | S. off. | S. car. | S. pra. | T. hyb. |
| --- | --- | --- | --- | --- | --- | --- | --- | --- |
| DCA axis 1 | + | + | + | + | + | + | + | + |
| DCA axis 2 | + | + | + | + | + | + | + | + |
| meadow/verge | + |  |  |  |  |  | + |  |
| log abund. A. syl. | + |  |  |  |  |  |  |  |
| log abund. C. jac. |  | + | + | + | + | + | + | + |
| log abund. H. spp. |  | + | + |  | + | + |  | + |
| log abund. R. acr. | + | + | + | + | + | + | + | + |
| log abund. S. off. | + | + | + | + | + | + | + | + |
| log abund. S. car. |  | + | + |  | + | + |  | + |
| log abund. S. pra. |  |  |  | + |  |  | + |  |
| log abund. T. hyb. | + | + | + | + | + | + |  | + |

Table S4 – Synoptic table of flowering plant species composition of the sectors at the study meadow. Species occurrences expressed as percentage of plots within the group a given species occurs in; plant species sorted according to decreasing overall frequency.

| Sector | 1 | 2 | 3 | 4 | 5 | 6 | verges |
| --- | --- | --- | --- | --- | --- | --- | --- |
| No. of plots | 22 | 15 | 12 | 25 | 8 | 11 | 10 |
|  |  |  |  |  |  |  |  |
| Species | Relative frequency (%) | | | | | | |
| *Trifolium repens* | 59 | 100 | 58 | 80 | 100 | 82 | 0 |
| *Trifolium hybridum* | 32 | 100 | 83 | 88 | 75 | 55 | 0 |
| *Plantago lanceolata* | 18 | 100 | 100 | 96 | 75 | 18 | 0 |
| *Ranunculus acris* | 55 | 60 | 58 | 56 | 100 | 82 | 0 |
| *Centaurea jacea* | 50 | 73 | 75 | 80 | 38 | 9 | 10 |
| *Achillea ptarmica* | 82 | 33 | 50 | 36 | 88 | 36 | 70 |
| *Sanguisorba officinalis* | 64 | 53 | 100 | 32 | 25 | 45 | 30 |
| *Lathyrus pratensis* | 32 | 80 | 75 | 64 | 88 | 0 | 0 |
| *Prunella vulgaris* | 32 | 40 | 50 | 76 | 75 | 64 | 0 |
| *Lotus corniculatus* | 9 | 67 | 50 | 92 | 38 | 0 | 10 |
| *Galium album* s.lat. | 0 | 67 | 67 | 88 | 13 | 18 | 0 |
| *Potentilla erecta* | 91 | 40 | 25 | 12 | 13 | 27 | 50 |
| *Alchemilla species* | 0 | 67 | 92 | 76 | 0 | 0 | 0 |
| *Pimpinella saxifraga* s.str. | 0 | 47 | 58 | 80 | 13 | 0 | 0 |
| *Trifolium pratense* | 18 | 47 | 17 | 56 | 38 | 36 | 0 |
| *Hypericum maculatum* | 0 | 53 | 42 | 72 | 0 | 0 | 10 |
| *Mentha arvensis* | 32 | 7 | 8 | 24 | 38 | 64 | 20 |
| *Ranunculus flammula* | 41 | 7 | 0 | 0 | 38 | 100 | 20 |
| *Crepis biennis* | 0 | 53 | 25 | 52 | 0 | 0 | 0 |
| *Selinum carvifolia* | 0 | 27 | 75 | 36 | 0 | 9 | 10 |
| *Cerastium holosteoides* | 0 | 13 | 50 | 32 | 0 | 36 | 0 |
| *Myosotis arvensis* | 9 | 7 | 0 | 28 | 38 | 45 | 0 |
| *Succisa pratensis* | 50 | 13 | 0 | 0 | 0 | 0 | 40 |
| *Serratula tinctoria* | 14 | 20 | 75 | 4 | 0 | 0 | 0 |
| *Vicia tetrasperma* | 0 | 27 | 8 | 44 | 0 | 0 | 0 |
| *Galium uliginosum* | 27 | 13 | 8 | 4 | 13 | 36 | 0 |
| *Achillea millefolium* agg*.* | 0 | 0 | 17 | 44 | 0 | 0 | 0 |
| *Leucanthemum vulgare* | 0 | 27 | 17 | 24 | 0 | 0 | 0 |
| *Cirsium palustre* | 23 | 13 | 0 | 0 | 13 | 9 | 10 |
| *Potentilla anserina* | 0 | 20 | 8 | 16 | 25 | 0 | 0 |
| *Daucus carota* | 0 | 7 | 8 | 28 | 0 | 0 | 0 |
| *Hypericum perforatum* | 0 | 0 | 17 | 16 | 0 | 27 | 0 |
| *Angelica sylvestris* | 0 | 0 | 8 | 0 | 0 | 18 | 60 |
| *Campanula patula* | 0 | 7 | 8 | 20 | 0 | 0 | 0 |
| *Epilobium species* | 0 | 0 | 8 | 8 | 13 | 18 | 10 |
| *Polygonum species* | 0 | 0 | 0 | 4 | 13 | 45 | 0 |
| *Heracleum sphondylium* | 0 | 27 | 0 | 8 | 0 | 0 | 0 |
| *Cirsium arvense* | 0 | 0 | 8 | 12 | 0 | 9 | 0 |
| *Lythrum salicaria* | 5 | 0 | 0 | 0 | 0 | 9 | 20 |
| *Lysimachia nummularia* | 0 | 0 | 0 | 0 | 38 | 9 | 0 |
| *Anagallis arvensis* | 0 | 0 | 0 | 16 | 0 | 0 | 0 |
| *Stellaria graminea* | 0 | 0 | 0 | 16 | 0 | 0 | 0 |
| *Lysimachia vulgaris* | 0 | 0 | 0 | 0 | 0 | 0 | 40 |
| *Leontodon hispidus* | 0 | 0 | 0 | 12 | 0 | 0 | 0 |
| *Gnaphalium sylvaticum* | 0 | 0 | 0 | 8 | 0 | 9 | 0 |
| *Trifolium dubium* | 0 | 0 | 8 | 4 | 0 | 0 | 0 |
| *Leontodon autumnalis* | 0 | 0 | 0 | 4 | 13 | 0 | 0 |
| *Scutellaria galericulata* | 0 | 0 | 0 | 0 | 0 | 0 | 20 |
| *Vicia hirsuta* | 0 | 7 | 0 | 0 | 0 | 0 | 0 |
| *Vicia sepium* | 0 | 7 | 0 | 0 | 0 | 0 | 0 |
| *Lychnis flos-cuculi* | 5 | 0 | 0 | 0 | 0 | 0 | 0 |
| *Odontites vernus* | 0 | 0 | 8 | 0 | 0 | 0 | 0 |
| *Cirsium canum* | 0 | 0 | 8 | 0 | 0 | 0 | 0 |
| *Polygonum aviculare* | 0 | 0 | 0 | 4 | 0 | 0 | 0 |
| *Medicago lupulina* | 0 | 0 | 0 | 4 | 0 | 0 | 0 |
| *Vicia cracca* agg. | 0 | 0 | 0 | 4 | 0 | 0 | 0 |
| *Lycopus europaeus* | 0 | 0 | 0 | 0 | 0 | 0 | 10 |
